# Downregulation of decidual SKP2 is associated with human recurrent miscarriage

**DOI:** 10.1186/s12958-021-00775-4

**Published:** 2021-06-11

**Authors:** Shijian Lv, Mei Liu, Lizhen Xu, Cong Zhang

**Affiliations:** 1 Center for Reproductive Medicine, Ren Ji Hospital, School of Medicine, Shanghai Jiao Tong University, Shanghai, 200135 China; 2 Shanghai Key Laboratory for Assisted Reproduction and Reproductive Genetics, Shanghai, China; 3 Department of Obstetrics, Affiliated Hospital of Shandong University of Traditional Chinese Medicine, No. 42 Wenhua Xi Road, Jinan, 250011 Shandong China; 4 Shandong Provincial Key Laboratory of Animal Resistance Biology, College of Life Sciences, Shandong Normal University, Jinan, Shandong China

**Keywords:** Recurrent miscarriage, Decidualization, SKP2, GLUT1

## Abstract

**Background:**

Recurrent miscarriage (RM) is a very frustrating problem for both couples and clinicians. To date, the etiology of RM remains poorly understood. Decidualization plays a critical role in implantation and the maintenance of pregnancy, and its deficiency is closely correlated with RM. The F-box protein S-phase kinase associated protein 2 (SKP2) is a key component of the SCF-type E3 ubiquitin ligase complex, which is critically involved in ErbB family-induced Akt ubiquitination, aerobic glycolysis and tumorigenesis. SKP2 is pivotal for reproduction, and SKP2-deficient mice show impaired ovarian development and reduced fertility.

**Methods:**

Here, we investigated the expression and function of SKP2 in human decidualization and its relation with RM. A total of 40 decidual samples were collected. Quantitative PCR analysis, western blot analysis and immunohistochemistry analysis were performed to analyze the differential expression of SKP2 between RM and control cells. For in vitro induction of decidualization, both HESCs (human endometrial stromal cells) cell line and primary ESCs (endometrial stromal cells) were used to analyze the effects of SKP2 on decidualization via siRNA transfection.

**Results:**

Compared to normal pregnant women, the expression of SKP2 was reduced in the decidual tissues from individuals with RM. After in vitro induction of decidualization, knockdown of SKP2 apparently attenuated the decidualization of HESCs and resulted in the downregulation of HOXA10 and FOXM1, which are essential for normal human decidualization. Moreover, our experiments demonstrated that SKP2 silencing reduced the expression of its downstream target GLUT1.

**Conclusions:**

Our study indicates a functional role of SKP2 in RM: downregulation of SKP2 in RM leads to impaired decidualization and downregulation of GLUT1 and consequently predisposes individuals to RM.

**Supplementary Information:**

The online version contains supplementary material available at 10.1186/s12958-021-00775-4.

## Background

Recurrent miscarriage (RM) is a distressing reproductive problem defined by two or more early miscarriages according to the guidelines of the American College of Obstetrics and Gynecology (ACOG) [1]. It is estimated that 2–5% of couples experience two clinical miscarriages, while approximately 1% of them experience three or more losses, and the risk of RM rises with increasing maternal age [2]. RM is associated with psychological morbidity and has often proven to be frustrating for both patients and clinicians.

Knowledge of the pathogenesis of RM has increased in the past few decades. Historically, recurrent miscarriage has been attributed to genetic, structural, infective, endocrine, immune, thrombophilic disorders, or unexplained causes [3]. Previous studies have demonstrated that the invasion of trophoblasts into the maternal decidua is a very important process for normal pregnancy. Abnormal placentation, particularly defective trophoblast invasion, is thought to cause RM in women [4]. Recent studies have shown that the decidua is essential for implantation and the maintenance of pregnancy. RM is associated with compromised endometrial decidualization, an obligatory transformation process for implantation and the development of a human embryo [5]. Decidualization is dependent on the convergence of cyclic adenosine monophosphate (cAMP) and progesterone signaling pathways, which drive integrated changes at both the transcriptome and proteome levels [6]. Decidual cells produce various characteristic growth factors and cytokines, such as insulin-like growth factor binding protein 1 (IGFBP1) and prolactin (PRL), which have been widely used as phenotypic markers of decidual cells [7, 8]. Decidualizing stromal cells acquire the unique ability to resist inflammatory and oxidative insults and to protect the placental semi-allograft against maternal immune responses [6]. Dysfunctional decidualization leads to implantation failure, miscarriage and other pregnancy-associated disorders [9, 10].

It is well known that enhanced glucose influx is critical for decidualization. Glucose can supply decidualized tissue with nutrients for biosynthesis [11]. In addition, glucose regulates the histone acetylation of gene promoters, such as PRL, IGFBP1 and FOXO1. Furthermore, low glucose inhibits decidualization [12]. Glucose utilization is mediated by glucose transporters (GLUTs, SLC2 family). In the human endometrium, GLUT1 is highly expressed, whereas other glucose transporters, including those involved in insulin-dependent glucose uptake (GLUT2, GLUT4, GLUT8), cannot be detected [13]. When *GLUT1* is knocked down in HESCs, the ability to take up glucose is inhibited, accompanied by impaired decidualization [14, 15].

SKP2, which is associated with Cullin-1, SKP1, and RBX1, forms an SCF-type E3 ubiquitin ligase complex [16]. Previous studies have shown that SKP2 plays an important role in governing cell cycle progression and cell survival by promoting the destruction of numerous tumor suppressor proteins, including P27, P21, P57, P130, and FOXO1 [17]. Furthermore, SKP2 is pivotal for the maintenance of fertility, and defects in SKP2 may underlie the pathogenesis of abnormal gamete production and premature ovarian failure during the reproductive life of women [18]. SKP2 facilitates cell survival by limiting DNA damage and apoptosis triggered by oxidative stress [19]. A correlation between SKP2 overexpression and elevated AKT1 activity has been reported in many carcinomas [20, 21]. Both ERK1/2 and AKT signaling, which mediate gene expression associated with decidualization and glucose uptake, are essential for human endometrial decidualization [22, 23]. These results suggest a positive regulatory role of SKP2 in decidualization. Despite great research efforts, the pathogenesis of RM related to defective decidualization is poorly understood. Therefore, the aim of this study was to investigate the role of SKP2 in decidualization, which will help clarify the pathogenesis of RM.

## Methods

### Decidual tissue collection

This study was approved by the Ethics Committee of Renji Hospital (No. 2018033004). Human decidual tissues were obtained from women with clinically normal pregnancies (terminated for nonmedical reasons, *n* = 20) and women with unexplained RM (*n* = 20). All the participants were Han ethnicity. The average age of women with normal pregnancies used as healthy controls (HC) was 29.0 ± 3.4 years, while the average age of patients with RM was 28.1 ± 4.5 years. The gestational ages when pregnancies were terminated in the HC group and RM group were 9.2 ± 1.3 weeks and 8.2 ± 2.3 weeks, respectively.

The exclusion criteria were as follows: (1) uterine anatomical malformation according to pelvic examination and ultrasound; (2) genetic abnormalities; (3) endocrine or metabolic diseases; (4) infection; and (5) other known causes. Written consent was obtained from all subjects before the collection of decidual tissues according to the guidelines of the Ministry of Public Health of China. Decidual tissues were collected immediately after suction and curettage, and blood was removed by scrubbing the decidua on cotton gauze. Collected tissues were then quickly frozen in liquid nitrogen and subsequently stored at − 80 °C before use.

### Western blot analysis

Briefly, total protein extracts were prepared from homogenized tissues or cultured cells with RIPA buffer containing protease inhibitors and a phosphatase inhibitor (cOmplete & PhosSTOP, Roche, Basel, Switzerland). Protein was quantified with an Enhanced BCA Protein Assay Kit (Thermo Fisher Scientific, Waltham, MA, USA). Equal amounts of protein were separated by SDS-PAGE before wet transfer onto nitrocellulose membranes (GE Healthcare Life Sciences, Pittsburgh, PA, USA). The membranes were blocked with 5% nonfat milk for 2 h at room temperature. After blocking, the membranes were incubated at 4 °C overnight with primary antibodies against SKP2 (1:1000; ab183039, Abcam, Cambridge, UK), GLUT1 (1:1000; 66,290–1, Proteintech, Rosemont, IL, USA), or β-actin (1:5000; sc47778, Santa Cruz, AR, USA), the latter of which was used as an internal control. Protein complexes were developed and observed with a chemiluminescent detection system (Millipore, Waltham, MA, USA) and a G-Box Chemiluminescence image capture system (Syngene, Cambridge, UK). The resulting bands were analyzed using Gel-Pro Analyzer software (Media Cybernetics).

### Quantitative PCR analysis

Total RNA was extracted from cells or tissues using an animal total RNA isolation kit (Foregene, Chengdu, China) according to the manufacturer’s instructions. The concentration and purity of RNA were assessed using a spectrophotometer (NanoDrop 2000c, Thermo Fisher Scientific, Waltham, MA, USA). Total RNA (500 ng) was reverse transcribed using a ReverTra Ace qPCR RT Kit (Toyobo, Osaka, Japan), and the resulting cDNA was used as the template for qPCR. Gene expression quantitation was performed in triplicate using SYBR GREEN PCR Master Mix (Toyobo, Osaka, Japan) in an Applied Biosystem Real-Time PCR system (Thermo Fisher Scientific, Waltham, MA, USA). *ACTB* was used as an internal control. The results were analyzed using the ΔΔCt method, and the sequences of the primers for PCR are listed in Table 1.

**Table 1:** Primer sequences

Gene		Sequences	Fragment size (bp)
***SKP2***	Forward Reverse	5′-ATGCCCCAATCTTGTCCATCT-3′ 5′-CACCGACTGAGTGATAGGTGT-3’	111
***IGFBP1***	Forward Reverse	5′-GGCACAGGAGACATCAGGAGAA-3′ 5′-GGTAGACGCACCAGCAGAGT-3′	131
***PRL***	Forward Reverse	5′-CATATTGCGATCCTGGAATGAG-3′ 5′-GATGAACCTGGCTGACTATCA-3′	158
***FOXO1***	Forward Reverse	5′-CTGGCTCTCACAGCAATGAT-3′ 5′-CACCATAGAATGCACATCCC-3′	146
***HOXA10***	Forward Reverse	5′-TCACCAAGGCCAGCACATAG-3′ 5′-TTAACTCAAGCTGCCTCGCC-3′	206
***FOXM1***	Forward Reverse	5′-GGAGGAAATGCCACACTTAGCG-3′ 5′-TAGGACTTCTTGGGTCTTGGGGTG-3′	150
***GLUT1***	Forward Reverse	5′-TGACCATCGCGCTAGCACTGC-3′ 5′-AACGGCAATGGCAGCTGGACG-3′	167
***ACTB***	Forward Reverse	5′-GGGAAATCGTGCGTGACATTAAG-3′ 5′-TGTGTTGGCGTACAGGTCTTTG-3′	275

### Immunohistochemistry

Immunohistochemistry was performed as previously described [24, 25]. Briefly, paraffin sections (5 μm) of human decidua from early pregnancy were dehydrated in a graded ethanol series and incubated with 3% hydrogen peroxide to block endogenous peroxidase. Subsequently, after antigen retrieval, the sections were incubated with 0.3% Triton X-100 for 20 min. The sections were then blocked with immunoglobulin G and incubated with a primary antibody against SKP2 overnight at 4 °C (1:50, ab183039, Abcam, Cambridge, UK). The sections were overlaid with peroxidase-conjugated goat anti-rabbit IgG (PV-9000, ZSGB-BIO, Beijing, China); then, the reaction was developed with 3,3′-diaminobenzidine and counterstained with hematoxylin. Negative controls were treated with nonimmune serum instead of the primary antibody.

### HESC culture and treatment

HESCs kindly donated by Dr. Haibin Wang (Xiamen University, Xiamen, China) were cultured in Phenol Red-free Dulbecco’s modified Eagle’s medium (DMEM)/F-12 plus 10% charcoal-stripped fetal bovine serum (Biological Industries) at 37 °C in a 5% CO_2_ atmosphere. To induce decidualization in vitro, HESCs were incubated with 1 μmol/L medroxyprogesterone-17-acetate (MPA) (Sigma-Aldrich, St. Louis, MS, USA) and 0.5 mmol/L N6,20-O-dibutyryladenosine cAMP sodium salt (db-cAMP) (Sigma-Aldrich, St. Louis, MS, USA). The medium was replaced every 2 days.

### Primary endometrial stromal cell (ESC) and primary decidual stromal cell (DSC) isolation

Proliferative endometrial tissues were obtained from women with normal menstrual cycles via endometrial biopsy at the time of diagnostic hysteroscopy. Primary ESCs were isolated according to previously described methods [26]. Briefly, endometrial tissues were fully washed with PBS and then minced in DMEM/F12. Minced tissues were digested with collagenase type I (c0130; Sigma-Aldrich, St. Louis, MS, USA) for 70 min at 37 °C and sequentially digested with deoxyribonuclease (DN25; Sigma-Aldrich, St. Louis, MS, USA) for 20 min at 37 °C. The suspension was filtered through 180 μm and 40 μm sterile griddles and centrifuged at 300×g for 5 min. The supernatant was discarded, and the cell pellet was suspended and inoculated in 6-well plates (10^6^ cells per well). Decidualization was induced according to the methods employed for HESCs [27].

Primary DSCs were isolated according to previously described methods [27]. Briefly, decidual tissues were obtained from women whose healthy pregnancies were artificially terminated by personal choice, then the samples were washed in PBS and minced in DMEM/F12.

### Cell transfection

Cultured cells, including HESCs and isolated primary ESCs, were transfected with siRNA using Lipofectamine 3000 Reagent (Thermo Fisher Scientific, Waltham, MA, USA). For in vitro-induced decidualization, cAMP and MPA were added after 24 h of transfection. The medium was removed and replaced every 2 days.

### Statistical analysis

All experiments were independently repeated at least three times. All data were analyzed using SPSS statistical software (Version 22.0, IBM, Chicago, Illinois, USA). Differences between two groups were analyzed by one-way ANOVA or Student’s *t*-test. All data are presented as the mean ± SEM. Differences were considered statistically significant at *P* < 0.05.

## Results

### SKP2 expression is decreased in the decidual tissues of RM patients

**Fig. 1:**
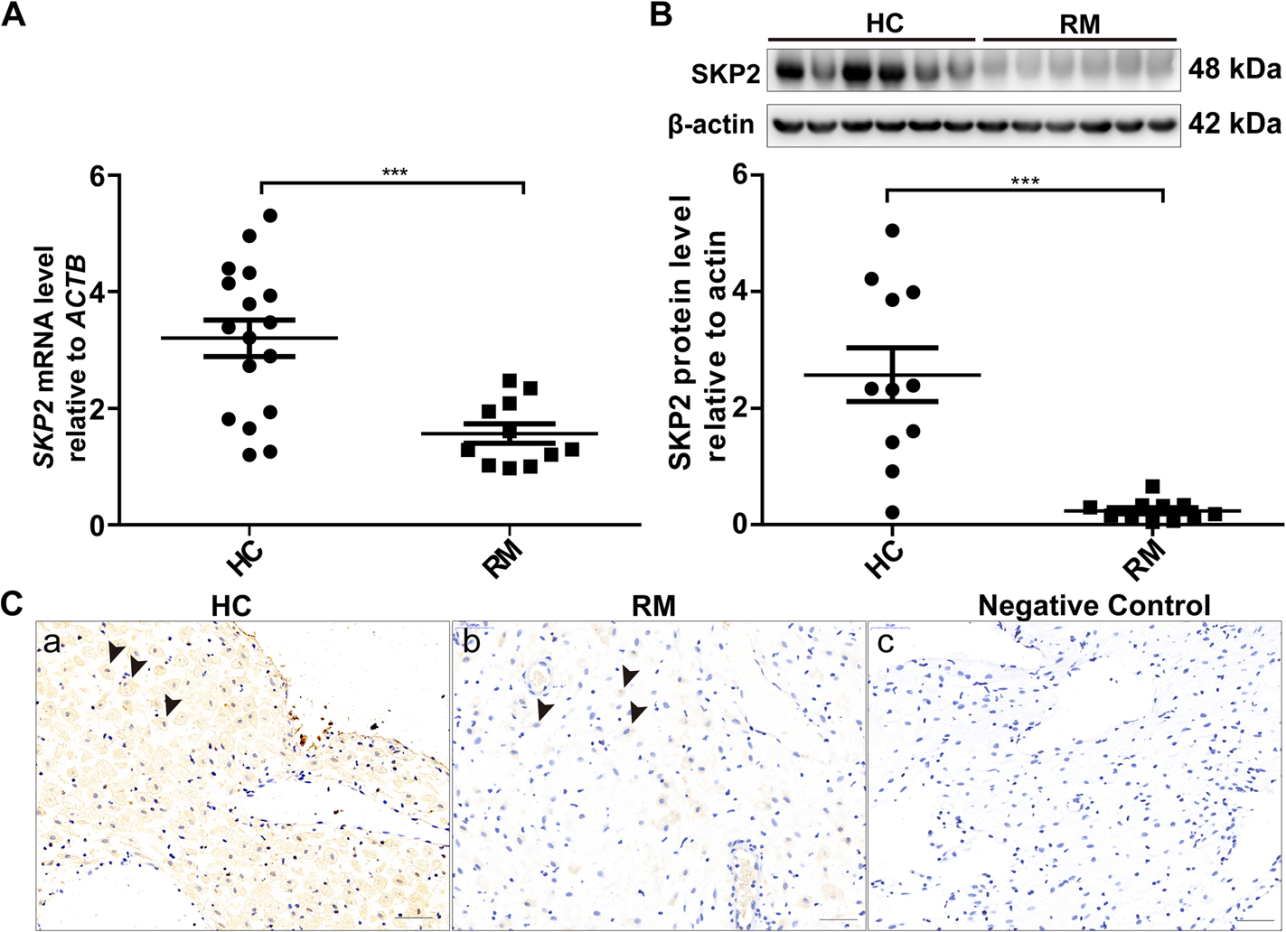
SKP2 expression is downregulated in decidual tissues of women with RM. **A** The *SKP2* mRNA levels in decidual tissues of RM patients (*n* = 20) and HCs (*n* = 20) were determined by qPCR. The relative amount of RNA was calculated using the 2^-∆∆Ct^ method and normalized with an internal control, *ACTB.* **B** The SKP2 protein levels relative to beta-actin were determined by western blot analysis (*n* = 11 for each group). The results are presented as the mean ± SEM. *p < 0.05. **p < 0.01. ***p < 0.001. **C** Localization of SKP2 in human decidual tissues. (a) Decidual sections from the HC group; (b) Decidual sections from the RM group; (c) Negative control. Brown staining represents the target protein. HC, healthy control; RM, recurrent miscarriage. Arrowheads indicate the decidual stromal cells. Scale bars = 50 μm

To determine whether SKP2 contributes to the pathogenesis of RM, we conducted qPCR and western blotting analyses of first trimester decidual tissues from RM and HC women. At both the mRNA and protein levels, SKP2 expression was significantly decreased in the decidual tissues of RM patients compared to that of HCs (Fig. 1A, B), thus indicating its possible role in decidualization. Immunohistochemistry staining of decidua sections further verified the decreased expression of SKP2 in tissues from RM patients (Fig. 1C).

### Knockdown of *SKP2* attenuates decidualization

**Fig. 2:**
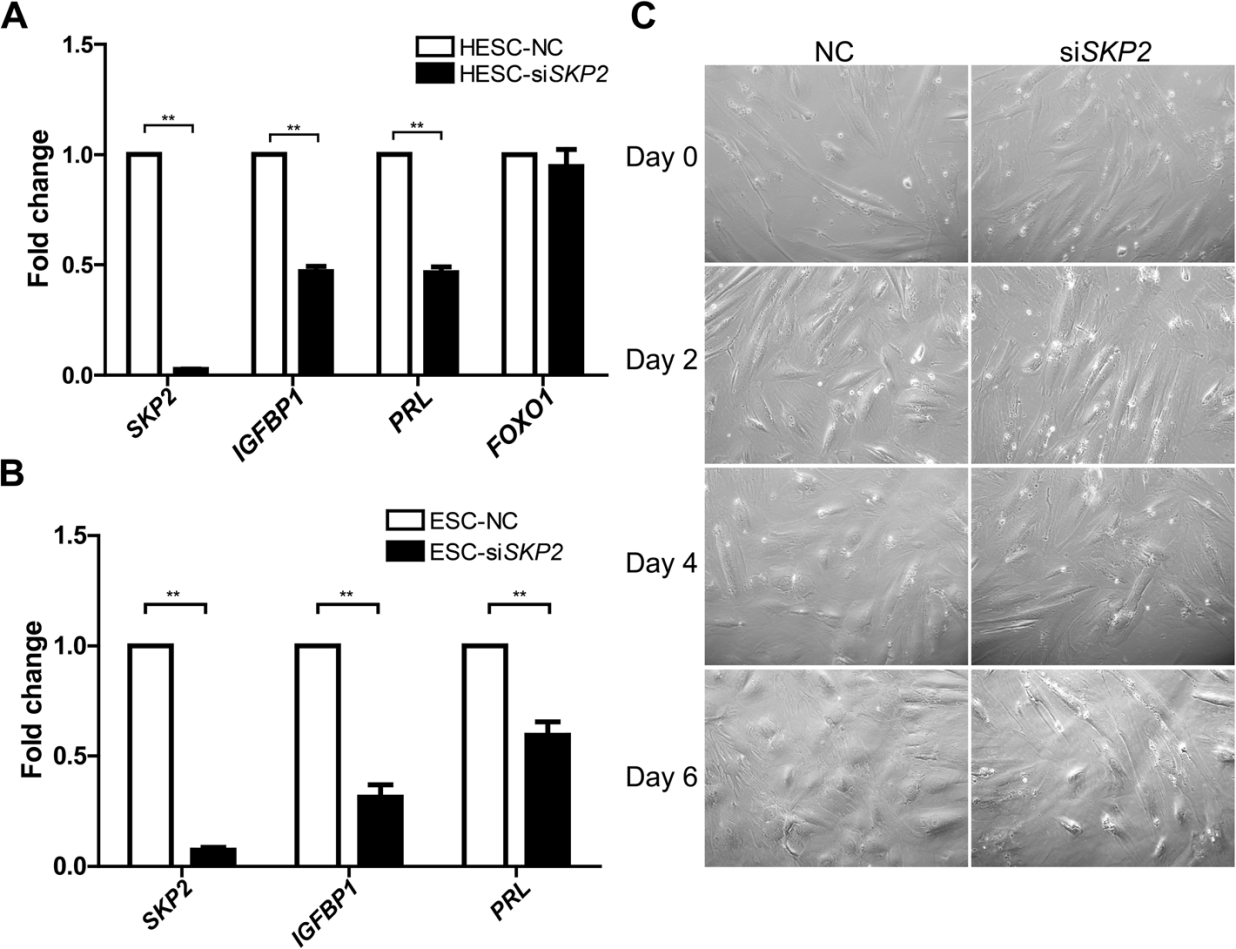
Effects of silencing *SKP2* on human decidualization. **A** *SKP2* knockdown caused impaired decidualization of HESCs. Decidualization markers (*IGFBP1* and *PRL*) decreased in HESCs, in which decidualization was induced for 4 days after transfection with si*SKP2* for 24 h. **B** The influence of *SKP2* knockdown on the decidualization of primary human ESCs. Decidualization was induced in primary human ESCs for 4 days after transfection with NC or si*SKP2*. **C** The influence of SKP2 knockdown on the cell morphology of HESCs after decidualization induction. Decidualization was induced with MPA and db-cAMP after transfection with NC or si*SKP2* for 24 h. HESCs (human endometrial stromal cells); ESCs (endometrial stromal cells); NC, scramble siRNA; si*SKP2*, siRNA against *SKP2*. All data are shown as the mean ± SEM. *P < 0.05; **P < 0.01

To examine how SKP2 affects the decidualization of the endometrium, si*SKP2* were transiently transfected into HESCs, which were then induced with db-cAMP and MPA for 4 days. Knockdown of SKP2 apparently attenuated the decidualization of HESCs, as verified by the reduced expression of *IGFBP1* and *PRL* during induction (Fig. 2A). However, SKP2 knockdown did not influence the transcription of FOXO1 (Fig. 2A). Similar results were obtained when SKP2 was knocked down of in primary ESCs isolated from proliferating endometria (Fig. 2B).

We also noted that SKP2 knockdown during decidualization caused differences in the morphology of db-cAMP and MPA induced HESCs: while the control cells were enlarged and spherical by induction 6 days, the morphology of the siSKP2 transfected cells remained fibroblast-like (Fig. 2C), clearly suggesting that silencing of SKP2 blocks the extensively-characterized morphological change of cells that occurs during decidualization.

**Fig. 3:**
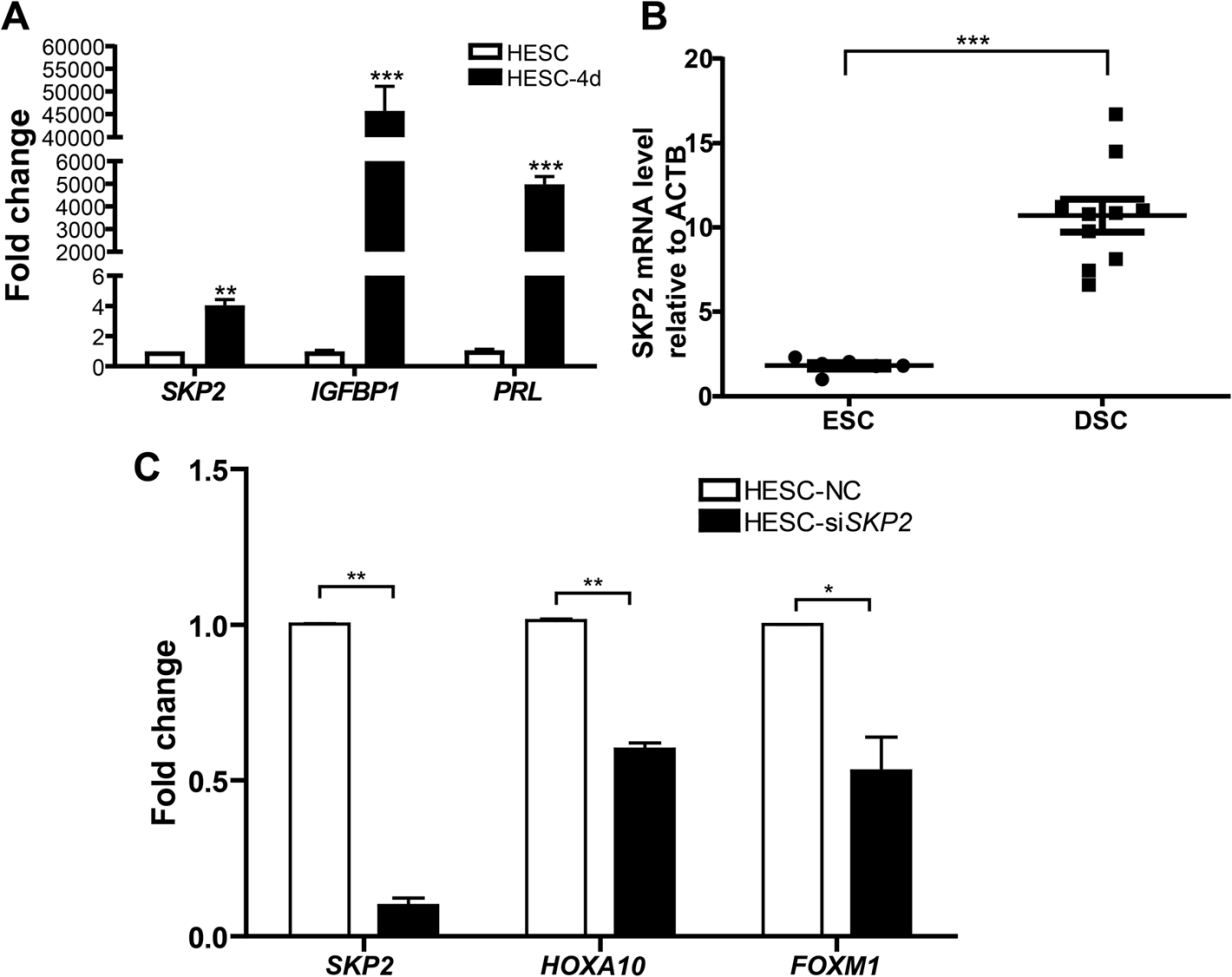
SKP2 is up-regulated during decidualization and its regulatory function in human decidualization. **A** The levels of *SKP2* mRNA was up-regulated in HESCs after the induction of decidualization. Decidualization was induced by the addition of MPA and db-cAMP for 4 days. **B** The expression of *SKP2* was analyzed by qPCR between decidualized stromal cells (DSCs) from first trimester pregnancy (*n* = 10) and endometrial stromal cells (ESCs) from proliferative endometria (*n* = 6). **C** Decidualization of HESCs was induced by the addition of MPA and db-cAMP for 4 days. The expression of the transcription factors *HOXA10* and *FOXM1* during decidualization was detected by qPCR. The levels of *HOXA10* and *FOXM1* mRNA were downregulated in HESCs during induced decidualization after *SKP2* knockdown. Data are shown as the mean ± SEM. *P < 0.05; **P < 0.01

Next, we used in vitro experiments with HESCs to confirm the function of SKP2 in the decidualization process. We induced the decidualization of HESCs using db-cAMP and MPA for 4 days and observed that the expression level of *SKP2* mRNA was significantly upregulated compared to uninduced control cells, accompanying with the significant increase in the mRNA levels of the decidualization biomarkers (*IGFBP1* and *PRL*) in the induced cells (Fig. 3A). Furtherly, we found that SKP2 expression at the mRNA level was significantly upregulated in primary DSCs isolated from first trimester decidual tissues as compared to primary ESCs derived from normal proliferative endometria (Fig. 3B).

To elucidate the mechanism underpinning SKP2-mediated regulation of decidualization, the transcriptional factors involved in decidualization were examined. As critical transcriptional factors, both HOXA10 and FOXM1 were found to be upregulated during decidualization [28, 29]. HESCs were transfected with siRNA against SKP2 and then induced with cAMP plus MPA for another 4 days. The results showed that both HOXA10 and FOXM1 were apparently decreased by the knockdown of SKP2 (Fig. 3C).

### SKP2 reduction attenuates GLUT1 expression during the process of decidualization

**Fig. 4:**
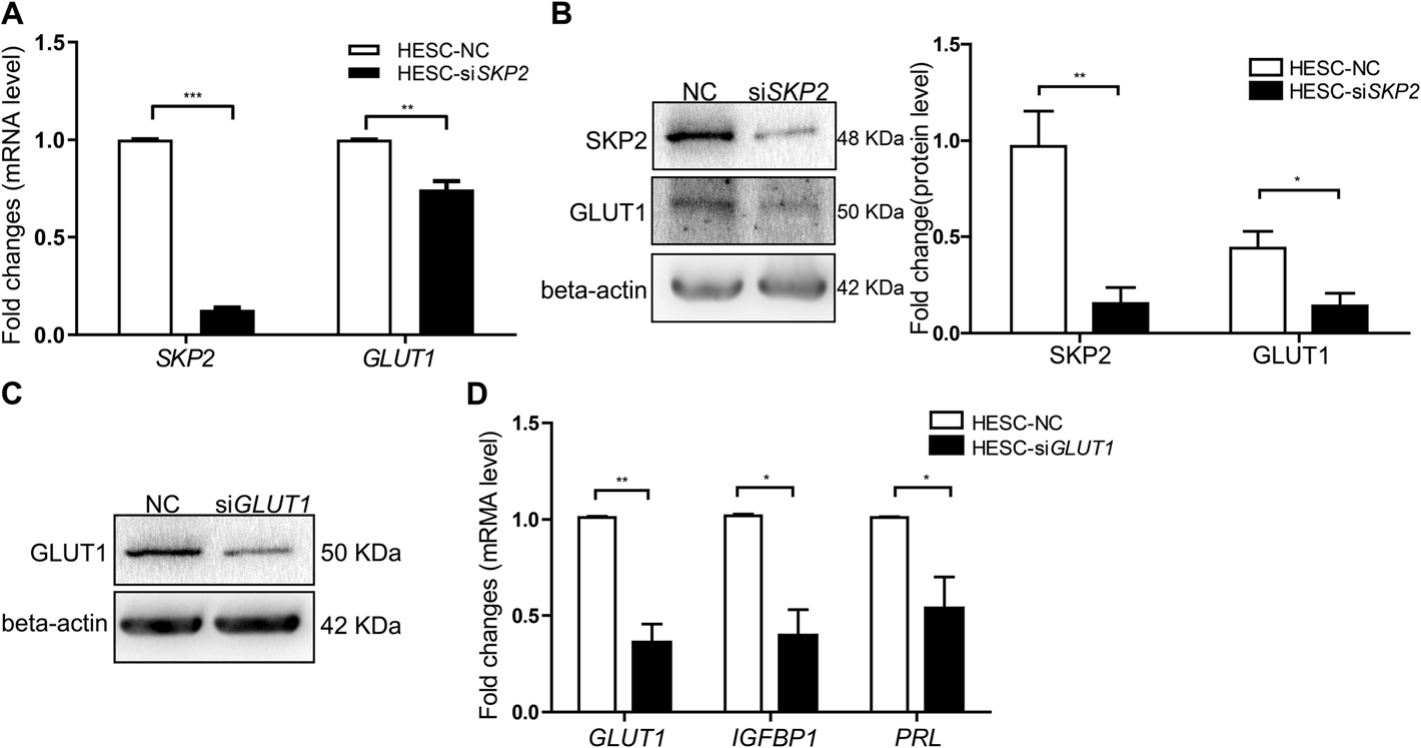
SKP2 silencing attenuates decidualization through downregulation of GLUT1 expression during induced decidualization. **A**, **B** HESCs transfected with scramble siRNA or si*SKP2* were incubated in induction medium for four days. Both mRNA and protein expression of GLUT1 were reduced after knockdown of SKP2. **C**, **D** GLUT1 silencing attenuated decidualization. Knockdown of GLUT1 in HESCs transfected with control siRNA or si*GLUT1* was determined by western blot analysis. mRNA expression of *GLUT1, IGFBP1,* and *PRL* was determined by qPCR. All data are shown as the mean ± SEM. *P < 0.05; **P < 0.01. ***P < 0.001

The decidualization of HESCs requires an increased supply of nutrients for biosynthesis, in which glucose metabolism plays important roles [13, 30]. The uptake of glucose, mediated by glucose transporters, is the first step of glucose utilization. Among these GLUT isoforms, GLUT1 is highly expressed and increased during decidualization [31]. To explore the effect of SKP2 on the expression of GLUT1, in vitro decidualization of HESCs after the knockdown of SKP2 was induced. The results demonstrated that GLUT1 expression was decreased in SKP2 transient silencing-based interference with decidualization (Fig. 4A, B), which indicated that SKP2 may increase the uptake of glucose mediated by GLUT1.

### The effect of GLUT1 knockdown on decidualization according to the expression of *PRL* and *IGFBP1*

To investigate the role of GLUT1 in decidualization, HESCs with knocked down GLUT1 were incubated with cAMP plus MPA for 4 days, and then, decidualization was evaluated by measuring the mRNA levels of *PRL* and *IGFBP1*. The results indicated that the mRNA levels of *PRL* and *IGFBP1* were remarkably decreased after GLUT1 knockdown (Fig. 4C, D). These results indicated that GLUT1 expression contributes to the expression of PRL and IGFBP1 in HESCs undergoing decidualization.

## Discussion

In this study, we elucidated the role of SKP2 during human decidualization. The expression of SKP2 at both the mRNA and protein levels significantly decreased in the decidua of women who suffered from RM compared to decidua from HC. In both HESCs and primary ESCs, silencing of *SKP2* inhibited decidualization. A mechanistic study demonstrated that SKP2 regulated human decidualization not only through critical transcriptional factors, such as HOXA10 and FOXM1, but also through the regulation of GLUT1. These findings demonstrated that SKP2 is critical for human decidualization and that its dysfunction in the decidua is associated with RM.

RM is a common and troublesome reproductive disorder. Emerging evidence indicates that abnormal decidualization contributes to the development of RM [32]. ESCs of women with RM show an abnormal response to decidualization in vitro, which is manifested by attenuated PRL production [33]. Decidualization is characterized by the secretory transformation of the uterine glands, the influx of specialized uterine natural killer cells and macrophages, and vascular remodeling [34]. The major secretory products of decidualization include PRL and IGFBP1. RM is associated with aberrant decidualization of human ESCs [35, 36]. These findings are supported by our study, which showed reduced expression of SKP2 in decidual tissues from the RM group and impaired decidualization caused by knockdown of *SKP2*.

Decidualization is crucial for embryo implantation and the maintenance of pregnancy. The core transcriptional factors that regulate decidual genes include FOXO1, FOXM1 and HOXA10 [37]. Both FOXO1 and FOXM1 belong to the large family of forkhead box transcription factors. The expression of FOXO1 is known to be induced during decidualization and required for human decidualized ESCs [38]. FOXO1 can interact with the progesterone receptor in decidualized human ESCs to control cell proliferation and epithelioid differentiation [39]. In addition, FOXO1 regulates uterine epithelial integrity and progesterone receptor expression, which are critical for embryo implantation [40]. Another member, FOXM1, is highly expressed in proliferating cells and plays pivotal roles in DNA replication and mitosis [41]. FOXM1 is essential for human stromal cell decidualization since uterine conditional deletion of FOXM1 reveals regional decidualization defects via impaired stromal cell mitosis and aberrantly upregulated polyploidy at the site of implantation [42]. HOXA10 belongs to the homeobox gene superfamily and is a well-known transcriptional regulator. HOXA10 plays an essential role during embryonic development and functional endometrial differentiation [43]. HOXA10 expression in the endometrium is significantly reduced in women with recurrent implantation failure and RM compared with fertile control women [29]. Other research has demonstrated that enhanced HOXA10 sumoylation inhibits embryo implantation in women with recurrent implantation failure [44]. Collectively, these transcriptional factors play crucial roles in decidualization. In the present study, SKP2 knockdown was accompanied by a decrease in the above-mentioned transcription factors, HOXA10 and FOXM1, which are regarded as being indispensable for human decidualization in other studies [38, 42]. We demonstrated that SKP2 plays a crucial role during decidualization since silencing of *SKP2* affected the decidualization of HESCs.

Apart from the morphological and biochemical reprogramming of the endometrial stromal compartment, the significance of metabolism in decidualization has been emphasized [12, 15]. Decidualization requires an increased supply of nutrients for biosynthesis, while glucose metabolism plays important roles. Glucose uptake is increased during decidualization, while decidualization is inhibited by incubating ESCs under low-glucose conditions [14]. Glucose plays a key role in decidualization by activating histones that include the promoters of *PRL, IGFBP1* and *FOXO1* [12]. Glucose metabolism is mediated by a family of glucose transporters. In the human endometrial stroma, only GLUT1 is expressed [31]; it is responsible for basal uptake and the storage of glucose, and endometrial mRNA transcription of GLUT1 increases progressively throughout gestation [45]. The downregulation of GLUT1 during decidualization by si*SKP2* was verified in the present study. Furthermore, when GLUT1 was knocked down in HESCs, impaired decidualization was confirmed. These results indicated a positive role of SKP2 in maintaining human decidualization through GLUT1. This finding is in line with the results of other research groups: SKP2 deficiency downregulates GLUT1 expression, likely through impairment of AKT1 activation [20].

## Conclusions

To summarize, our results suggest that SKP2 is very important in the maintenance of pregnancy by promoting decidualization during early pregnancy. Reduced expression of SKP2 in decidual tissues is associated with RM through compromised decidualization. Therefore, our study of SKP2 in human decidualization provides new insights into the mechanisms and management of RM.

## Supplementary Information

**Additional file 1.**

## Data Availability

All data generated through this study are included in this article.
