# Supplementary figures and images for: Downregulation of decidual SKP2 is associated with human recurrent miscarriage
Source: Reprod Biol Endocrinol. 2021 Jun 11;19:88. doi: 10.1186/s12958-021-00775-4 (PMC8194034; doi:10.1186/s12958-021-00775-4)

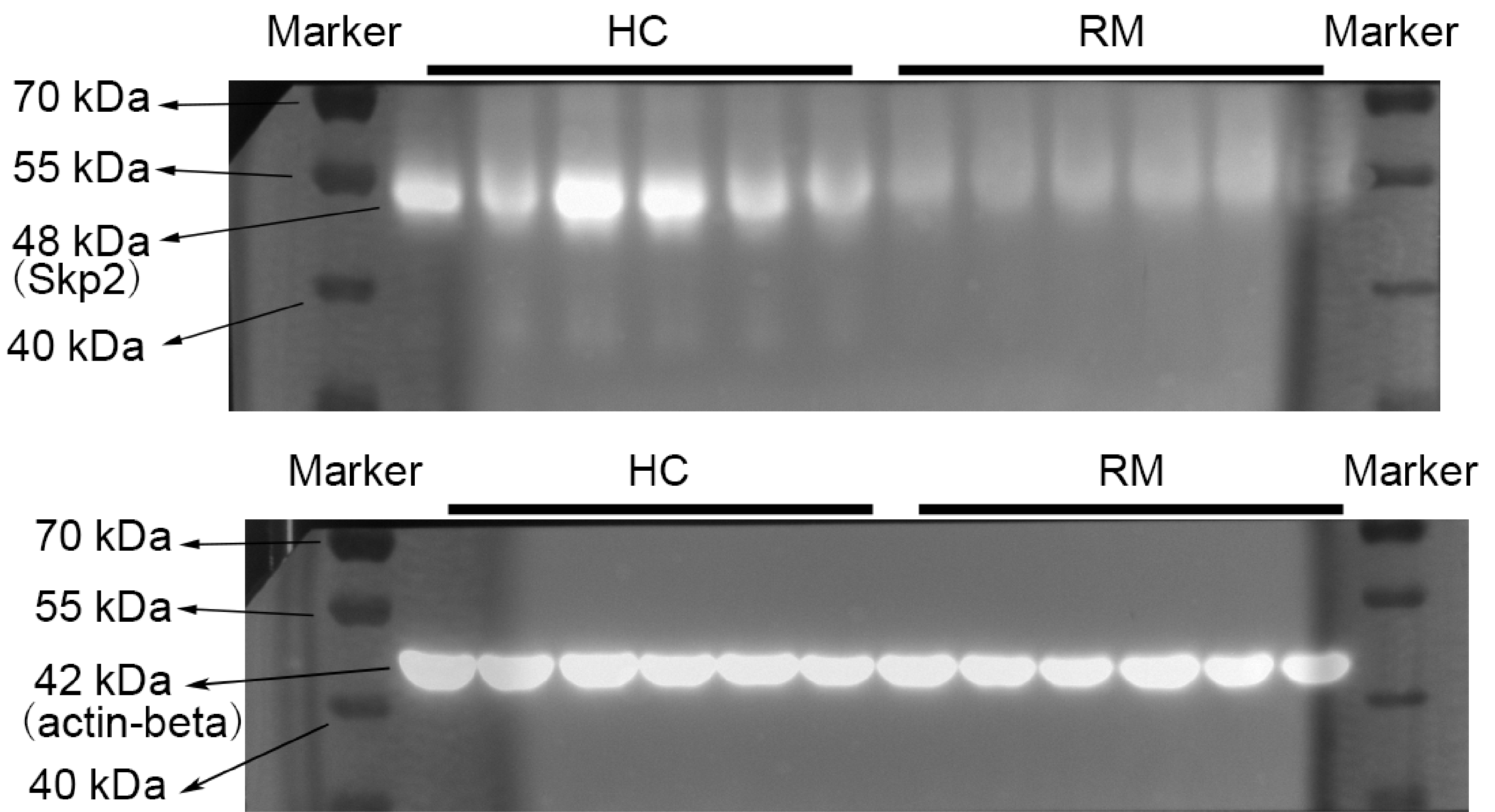

Supplement: Supplementary file 1 — Additional file 1. [file 12958_2021_775_MOESM1_ESM.tif]
